# Parent-Child Discrepancies in Perceived Parent-Child Communication and Depressive Symptoms in Early Adolescents in China

**DOI:** 10.3390/ijerph182212041

**Published:** 2021-11-16

**Authors:** Qiongwen Zhang, Daniel T. L. Shek, Yangu Pan

**Affiliations:** 1 Research Institute of Social Development, Southwestern University of Finance and Economics, Chengdu 611130, China; ilxpw@swufe.edu.cn; 2 Department of Applied Social Sciences, The Hong Kong Polytechnic University, Hong Kong 999077, China; daniel.shek@polyu.edu.hk

**Keywords:** early adolescence, depressive symptoms, parent-child communication, parent-child discrepancies, two-parent families, single-parent families, Chinese

## Abstract

Although recent studies demonstrated that parent-child discrepancies in the perceived family processes were associated with children’s developmental outcomes, few studies have addressed this issue in different types of families in mainland China. The present study investigated that how discrepancies in parents’ and adolescents’ perceptions of parent-adolescent communication were associated with early adolescent depressive symptoms in a nationally representative sample (*N* = 15,377) with 7010 father-adolescent dyads (adolescents: *Mage* = 14.24 years, *SD* = 1.25 years; 5960 adolescents from two-parent families, 443 adolescents from single-father families) and 8367 mother-adolescent dyads (adolescents: *Mage* = 14.02 years, *SD* = 1.18 years; 6670 adolescents from two-parent families, 1362 adolescents from single-mother families) in China. Adolescent respondents completed a measure of depressive symptoms and all informants reported on the perceived levels of parent-adolescent communication. Results indicated that adolescents reported parent-child communication more negatively than did their parents. Father-adolescent discrepancies were also greater in intact families than non-intact families. Polynomial regression analyses indicated that while there was a significant interactive effect of father-reported and adolescent-reported father-adolescent communication in Chinese two-parent families, no significant interaction was found for mother-adolescent dyad. Besides, adolescent-reported mother-child communication interacted with mother-reported communication in Chinese single-mother families only. The findings clarify parent-adolescent discrepancies in parent-child communication in different types of families in China and they have theoretical and practical implications on the role of discrepancies in parents and adolescent children on perceived parent-adolescent communication in early adolescent depressive symptoms.

## 1. Introduction

Compared to children, early adolescents face greater challenges and stresses arising from significant biological, cognitive, and social-emotional developmental changes [1,2,3], resulting in mental health issues such as internalizing and externalizing problems [4,5]. For example, high prevalence rates of adolescent depressive symptoms in the United States (13.3%) and China (24.6%) have been reported [6,7]. As many studies have demonstrated that depressive symptoms have detrimental impacts on adolescents’ concurrent and later psychosocial functioning [8,9], it is very important to identify factors influencing the risk of depressive symptoms during early adolescence.

Parent-adolescent communication as a key dimension of attachment plays an important protective role in shaping adolescent depressive symptoms [10,11,12]. Generally speaking, while positive parental communication was negatively associated with adolescent depressive symptoms [10,11,13], low quality parental communication such as rejection and criticism from parents was linked to higher parent-adolescent conflicts and stresses that contribute to depressive symptoms [14,15,16]. Previous studies also showed that paternal communication and maternal communication have different influence on adolescent depressive symptoms [17,18,19]. However, as parents and their adolescents often have different perceptions of parental communication, inconsistent findings in research and clinical settings were found [20,21].

There are studies suggesting that parent-child discrepancies in the perceived family processes could predict adolescent psychosocial adjustment [22,23,24]. For example, Kapetanovic and Boson [25] found that more positive parental perception of parent-child communication relative to adolescents’ reports was related to poorer adolescent mental health. However, it is noteworthy that except for a few studies (Shek’s studies on Chinese adolescents experiencing economic disadvantage) [26,27,28], not much effort has been spent to investigate the differential contribution of father-adolescent discrepancies and mother-adolescent discrepancies in parental communication to adolescent depressive symptoms in non-Western contexts. Therefore, this study attempted to examine how father-adolescent and mother-adolescent discrepancies in parent-child communication were associated with Chinese early adolescents’ depressive symptoms in two-parent and single-parent families.

### 1.1. Discrepancies in Perception of Parent-Child Communication between Parents and Adolescents

Both parents and children are important informants about parent-child communication. However, they might report different perceptions of the same construct [20,29,30], with parents often showing more positive perceived parental communication than their children [31,32]. In the scientific literature, there are two views on the nature and impact of parent-adolescent discrepancies in parent-adolescent communication.

From the normative development perspective, parent-adolescent discrepancies in parental communication are considered to be part of normal adolescent development [33,34]. Although early adolescents require more autonomy and independence [35,36], their parents may not adopt more open communication and have more active discussions to meet their children’s needs in a timely manner. In particular, when adolescents consider equal communication as an indication of autonomy and independence, but they cannot successfully employ communication and perspective-taking skills to handle parent-adolescent discrepancies in autonomy and independence [37], they may perceive parent-adolescent communication as less positive. Besides, the generational stake theory suggests that parents as nurturers of a generational bond may have a tendency to exaggerate the perception of positive communication, while adolescents are more likely to demonstrate their autonomy by disagreement with their parents [38,39].

Alternatively, from a pathological perspective, greater parent-adolescent discrepancies in parental communication were linked to greater problems in parent-adolescent relationships [40,41]. Theoretically, adolescents are less likely to share their thoughts and discuss conflicts when they have a low quality of parent-adolescent relationships characterized by alienation and coldness, because they believe that they cannot get emotional comfort and instrumental support through communication from their parents. As a result, they might perceive parental communication less positively than do their parents.

### 1.2. Parent-Adolescent Discrepancies in Parental Communication and Adolescent Depressive Symptoms

Although some researchers argued that discrepant reports between informants often were attributed to reporter bias and measurement errors [42], others have suggested that parent-adolescent discrepancies may provide important information about the nature of adolescent development within the family context [43,44]. Kapetanovic and Boson [25] reported that parent-adolescent discrepancies in parent-adolescent communication were related to adolescent psychological problems. Theoretically, attachment theory provides a comprehensive perspective to understand the association between discordance of communication between parents and their children and adolescent depressive symptoms [40,45]. Securely attached adolescents reported that their parents often encouraged them to communicate their emotions openly [46]. As a result, they are more likely to communicate openly and discuss problems with their parents [37,47], resulting in a low discrepancy in perceived parent-child communication. Moreover, securely attached adolescents may get more timely support from their parents through open communication that would decrease the risk of developing depressive symptoms [48,49]. In contrast, adolescents with an insecure attachment are less willing to communicate with their parents, which would lead to a discrepancy in perceived parent-adolescent communication [40]. Besides, parents’ overestimation of parent-adolescent communication will lead to a higher risk of depressive symptoms [25], because parents are unable to provide effective and timely support based on limited information.

### 1.3. Chinese Family Processes with Reference to Parent-Child Discrepancies in Parent-Adolescent Communication

In traditional Chinese culture, parental control is very strict. Shek [49] argued that traditional Chinese parental control included psychological control (expectation of total obedience of the child) and behavior control (high expectation and strict discipline). Relative to Western parents, Chinese parents are less likely to show their love through open communication, and Chinese adolescents are less willing to discuss disagreement with their parents openly [50]. As a result, Chinese parents and their children may perceive more discordance in parent-adolescent communication.

Moreover, in traditional Chinese families, fathers and mothers play different roles in parenting. This is reflected in the Chinese saying “men take care of things outside the family whereas women take care of things inside the family” (nan zhu wai, nv zhu nei). A father is seen as the head of a family and a mother is expected to take care of the basic needs of the children. Fathers were expected to play the role of teachers supervising the children to ensure that they behaved well, as there is a saying, “it is the fault of the father if he only raises the child without teaching him” (yang bu jiao, fu zhi guo) [51]. Thus, in traditional Chinese culture, fathers are expected to give more behavioral control and moral guidance to their children, and children are required to be more obedient. Finally, father and their adolescent children rarely negotiate disagreement and share decision-making, leading to greater discrepancies in father-adolescent communication. Furthermore, a recent study found that mothers showed higher levels of parental control and parent-child relational quality than did fathers during their high school years in Hong Kong [52]. Another study found that Chinese adolescents perceived maternal parenting attributes were more positive than perceived paternal parenting attributes, and this effect was found only among adolescent girls [53]. These findings suggest that father-child discrepancies in perceived father-adolescent communication are greater than mother-child discrepancies in perceived mother-adolescent communication in China. Unfortunately, despite recent work on Chinese families and adolescent development [54,55], studies on parent-adolescent discrepancies in perceived family processes are few.

Furthermore, due to industrialization, urbanization, and population migration, marriage and family have undergone profound changes in China, one of which is characterized by the diversification of family structure or living arrangement pattern. First, China’s crude divorce rate rose from 0.9 per 1000 people in 2002 to 2.79 in 2015 [56,57]. Second, intergenerational parenting is very common in China. A considerable number of couples send their children to grandparents’ homes for long periods of time, and the proportion of such “generational separation families” is increasing year by year [58]. Third, restricted by the household registration system and their own economic conditions, most rural migrant workers leave their children in their rural hometowns, resulting in a large number of “left-behind children” [59].

According to the living arrangement of children and parents, the Chinese family structure can be divided into four categories: two-parent families, single-mother families, single-father families, and both parents absent families (such as parents working in places remote from their home). Different types of families may have different characteristics regarding family processes. Shek [49] found that parental behavioral control and parent-child relational quality were poorer, while parental psychological control was higher, in non-intact families compared with intact families in China. In addition, Shek et al. [60] found that family functioning, parental behavioral control, paternal psychological control, parent-child relational qualities were poorer in non-intact families than intact families in China. These findings suggest parenting processes were poorer in non-intact families (such as parent-absent families and single-parent families) compared with those in two-parent families (i.e., intact families).

Although there is a growing concern for the influence of parent-child discrepancy in the perceived family process on adolescent psychological outcomes during the past decade [21,23,31], few studies investigated whether the parent-child discrepancies in parent-adolescent communication were related to adolescent depression, particularly in different Chinese societies. The related findings could clarify whether parent-child discrepancy in the perceived family process is normative or pathological and whether it is a measurement error or it has theoretical significance. Moreover, few studies investigated whether the relationship between parent-child discrepancies in parent-child communication and adolescent depression differ in Chinese families with different structures.

Methodologically, studies on parent-child discrepancies in the perceived family process in China was very few, except a few studies in Hong Kong [26,27,28,61,62]. Moreover, the sample size in previous studies on parent-child discrepancies was not large. Furthermore, few studies include both father-child and mother-child discrepancies. Finally, as previous studies demonstrated that polynomial regression analyses were superior in examining the relationship between informant discrepancies and adolescent developmental outcomes relative to difference scores [63,64], it is desirable to use this approach.

### 1.4. Research Questions in the Present Study

The present study aimed to answer three questions:

Research Question 1: Are there any differences in the perceptions of parent-adolescent communication between fathers or mothers and adolescents in China? Based on the existing literature [27,64], we predicted that there would be differences in the perceptions of parent-communication between fathers or mothers and adolescents, with adolescents having more negative perceptions than fathers or mothers.

**Hypothesis** **1a.** *There would be differences in the perceptions of parent-communication between fathers and adolescents, with adolescents having more negative perceptions than fathers.*

**Hypothesis** **1b.** *There would be differences in the perceptions of parent-communication between mothers and adolescents, with adolescents having more negative perceptions than mothers.*

Research Question 2: Is parent-adolescent discrepancy in parent-child communication different in families with different structures? Roughly speaking, there are four types of families under focus, including two-parent families (i.e., both parents are present), single-mother families, single-father families, and parent absent families (i.e., both parents are not at home). While the first one can be regarded as intact families, the latter three types are non-intact families. As family processes were more positive in intact families than non-intact families [60], we predicted that discrepancies in parent-adolescent communication would be greater in non-intact families than intact families.

**Hypothesis** **2.** *The discrepancies in parent-adolescent communication would be greater in non-intact families than intact families.*

Research Question 3: Are father-child and mother-child discrepancies in perceived parent-child communication related to adolescent depressive symptoms in four types of Chinese family structure, i.e., two-parent families, single-mother families, single-father families, and families with both parents absent, respectively?

For two-parent families, as fathers occupy more power in Chinese families [18,65], a parent-child discrepancy in parent-adolescent communication is disturbing for adolescents, particularly when fathers perceived high level, but adolescents perceived low of communication (i.e., the disappointment of mismatch). Hence, we hypothesized interactions between fathers’ and adolescents’ perceptions of father-child communication on adolescents’ depressive symptoms were significant in two-parent families. Specifically, high father-adolescent communication perceived by adolescents coupled with low father-adolescent communication perceived by fathers would be associated with a higher level of depression than did the condition when high levels of parent-adolescent communication were perceived by both parties.

**Hypothesis** **3.** *The interactions between fathers’ and adolescents’ perceptions of father-child communication on adolescents’ depressive symptoms were significant in two-parent families. Specifically, high father-adolescent communication perceived by adolescents coupled with low father-adolescent communication perceived by fathers would be associated with a higher level of depression than did the condition when high levels of parent-adolescent communication were perceived by both parties.*

In contrast, we did not propose this hypothesis for mother-adolescent communication in two-parent families.

On the other hand, as mothers in single-mother families have power and are responsible for the basic socialization tasks (i.e., frequent contacts), discrepant perceptions of parent-adolescent communication would be disturbing for adolescents. Hence, we hypothesized interactions between mothers’ and adolescents’ perception of mother-child communication on adolescents’ depressive symptoms in single-mother families. In particular, low mother-adolescent communication perceived by adolescents coupled with high mother-adolescent communication perceived by mothers (i.e., “beauty” on the mother’s side) would be associated with the highest level of depression.

**Hypothesis** **4.** *The interactions between mothers’ and adolescents’ perception of mother-child communication on adolescents’ depressive symptoms were significant in single-mother families. In particular, low mother-adolescent communication perceived by adolescents coupled with high mother-adolescent communication perceived by mothers (i.e., “beauty” on the mother’s side) would be associated with the highest level of depression.*

No similar hypothesis was made for single-father families because fathers are typically more detached in the socialization process.

## 2. Materials and Methods

### 2.1. Participants and Procedure

We used the nationally representative data from the China Education Panel Survey (CEPS) collected during September 2013 and March 2014. A total of 19,487 adolescents and their mothers (or fathers) from 438 classes in 112 middle schools in 28 counties (districts) in China completed the child-reported and parent-reported questionnaires, respectively.

In the present study, we deleted 175 participants whose depressive symptom scores were the maximal value to ensure normality of distribution. Additionally, we deleted 3176 participants with missing data. Finally, there were 15,377 parent-adolescent dyads (adolescents: *M*age = 14.12 years, *SD* = 1.22 years; *N*boys = 7677, *N*girls = 7700; *N*grade_7_ = 8060, *N*grade_9_ = 7317) participating in the study, including 7010 father-adolescent dyads and 8367 mother-adolescent dyads. Only one parent (father or mother) in a family participated. Please see Table 1.

### 2.2. Measures

#### 2.2.1. Father-Adolescent and Mother-Adolescent Communication

Following previous studies [10,12], we measured paternal and maternal communication as perceived by adolescents, fathers, and mothers with five items used to measure father- and mother-adolescent communication as perceived by adolescents, fathers, and mothers. Adolescents were asked, “How often did your father/mother have a discussion with you on the following issues?” The five response items are “What happened at school,” “Your relationship with your friends,” “Your relationship with your teacher,” “Your feelings,” and “Your mind or troubles.” Similarly, we assessed father- and mother-adolescent communication by fathers or mothers using the same five items (except replacing “your” by “child’s”), and fathers or mothers were asked, “How often did you have a discussion with your child on the following issues?” All items were rated on a three-point response scale (1 = never, 2 = once in a while, and 3 = often). In this study, internal consistency for parent-adolescent communication was found to be good across informants. Specifically, Cronbach’s α was 0.83 for adolescent reports on the mother-child communication, 0.83 for adolescent reports on the father-child communication, 0.87 for mother-reports on the mother-child communication, and 0.85 for father-reports on the father-child communication.

#### 2.2.2. Depressive Symptoms

According to previous measurement [66,67], depressive symptoms was measured by five items, including “depressed”, “upset”, “unhappy”, “sad”, and “life has no meaning”. Following the question “Have you had any of the following feelings in the past seven days?”, all items were rated on a five-point Likert-type scale ranging from 1 (almost never) to 5 (almost always). The previous study has demonstrated this measurement was valid in Chinese adolescents [19]. Cronbach’s α in this study was 0.85.

#### 2.2.3. Data Analyses

Regarding Research Question 1, paired *t* test was used to investigate father/mother-adolescent differences in perceived parent-child communication. Cohen’s d was used to determine the effect size [68]. Regarding Research Question 2, an independent-samples *t* test was used to examine whether parent-adolescent discrepancy in parent-child communication was different between intact families and non-intact families.

Regarding Research Question 3, polynomial multiple regression analyses [69] were used to test the interaction effect of father-reported (or mother-reported) and adolescent-reported parent-adolescent communication on adolescent depressive symptoms. The polynomial multiple regression model, used in testing parent and adolescent discrepancies of perceived parent-adolescent communication on the prediction of adolescent developmental outcomes, is shown in the equation:

*Y* = *b*_0_ + *b*_1_*A* + *b*_2_*P* + *b*_3_*A*^2^ + *b*_4_*AP* + *b*_5_*P*^2^ + *e* (1)

while *P* and *A* represent mothers’/fathers’ and adolescents’ reports of parent-adolescent communication, *b*_2_ and *b*_5_ represent the linear and quadratic effects of mother/father reports at the mean level of adolescent reports. Besides, *b*_1_ and *b*_3_ represent the linear and quadratic effects of adolescent reports at mean levels of mother/father reports, and *b*_4_ represents the interaction term between mother/father and adolescent reports. To avoid the misrepresentation of the quadratic effect of mother/father or adolescent reports by the interaction term, *A*^2^ and *P*^2^ were included in the equation [70,71]. We standardized the scores of parent-child communication. Simple slope analysis and plotting were used when interaction was significant [72]. Moreover, analyses were conducted separately for the mother-child and father-child communication. Additionally, analyses were conducted separately in four types of family structure, including two-parent families, single-father families, single-mother families, parent-absent families.

## 3. Results

Regarding Research Question 1, paired *t*-test analyses among total sample showed that there was a significant mean difference between adolescents’ reports (*M* = 11.11, *SD* = 2.54) and mothers’ reports (*M* = 11.87, *SD* = 2.54) of mother-adolescent communication, *t* = −25.62, *p* < 0.001, Cohen’s *d* = −0.28, which is small to medium effect size (Cohen, 1988). Similarly, adolescents reported (*M* = 9.77, *SD* = 2.85) less positive father-adolescent communication than did fathers (*M* = 11.23, *SD* = 2.52), *t* = −40.85, *p* < 0.001, Cohen’s *d* = −0.54, which is considered as medium to large effect size. Hypothesis 1a and Hypothesis 1b were supported. The findings can be seen in Table 2.

Regarding Research Question 2, results showed that parent-child discrepancy in perceived mother-child communication was not different between intact families (*M* = 0.76) and non-intact families (*M* = 0.76), *t* = −0.008, *p* = 0.994, whereas parent-child discrepancy in perceived father-child communication was larger in intact families (*M* = 1.51) than non-intact families (*M* = 1.21), *t* = 2.98, *p* = 0.003. The findings can be seen in Table 3. Hypothesis 2 was not supported.

Regarding Research Question 3, polynomial regression analyses were used to test the interaction effect of father-reported (or mother-reported) and adolescent-reported father-adolescent/mother-adolescent communication on adolescent depressive symptoms.

**Figure 1:**
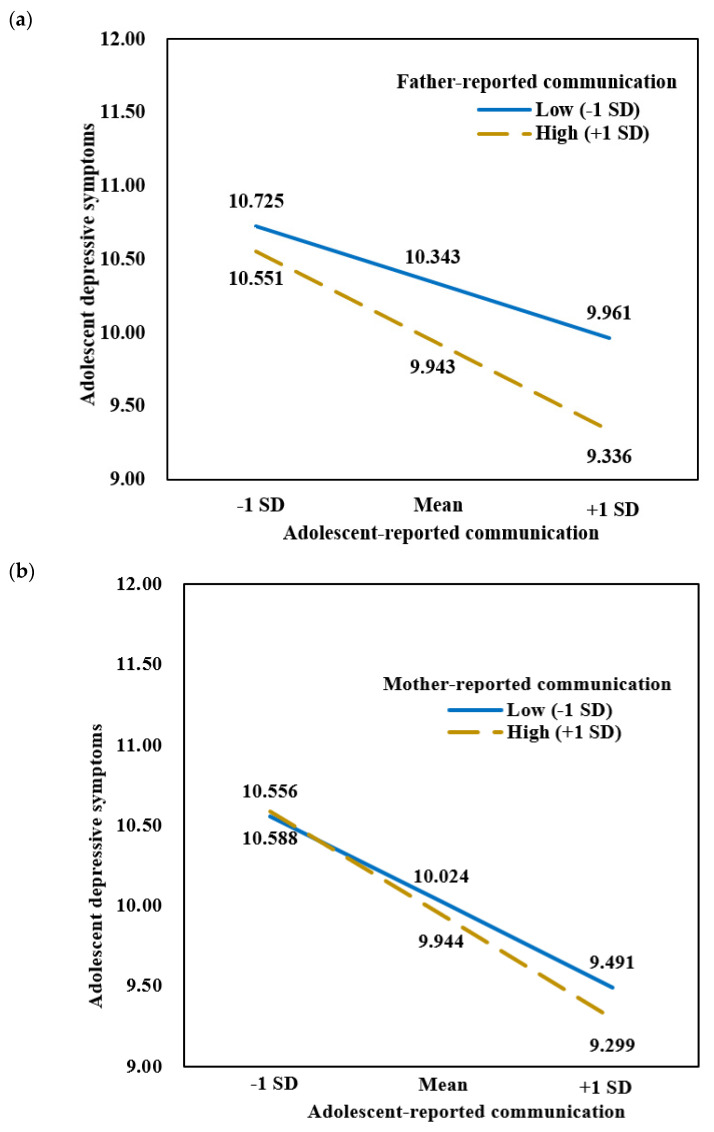
(**a**) Predicted values of depressive symptoms as a function of adolescent-reported communication at high and low levels of father-reported communication among adolescents in two-parent families (*N* = 5960). (**b**) Predicted values of depressive symptoms as a function of adolescent-reported communication at high and low levels of mother-reported communication among adolescents in two-parent families (*N* = 6670).

Results showed that in two-parent families, the interaction between father-reported and adolescent-reported father-adolescent communication on adolescent depressive symptoms was significant, *b* = −0.122, *SE* = 0.059, *t* = −2.068, *p* = 0.039, 95% *CI* = [−0.237, −0.006]. The findings are presented in Table 4. Simple slope analysis (see Figure 1a) indicated that adolescent-perceived father-child communication was more strongly linked with less adolescent depressive symptoms when father-perceived father-child communication was high, *b* = −0.625, *SE* = 0.077, *t* = −8.132, *p* < 0.001, 95% *CI* = [−0.775, −0.474] than when it was low, *b* = −0.393, *SE* = 0.079, *t* = −4.943, *p* < 0.001, 95% *CI* = [−0.549, −0.237]. Although similar pattern was found for mother-adolescent communication discrepancies in two-parent families, the interaction was not significant (see Figure 1b). This provides support for Hypothesis 3.

**Figure 2:**
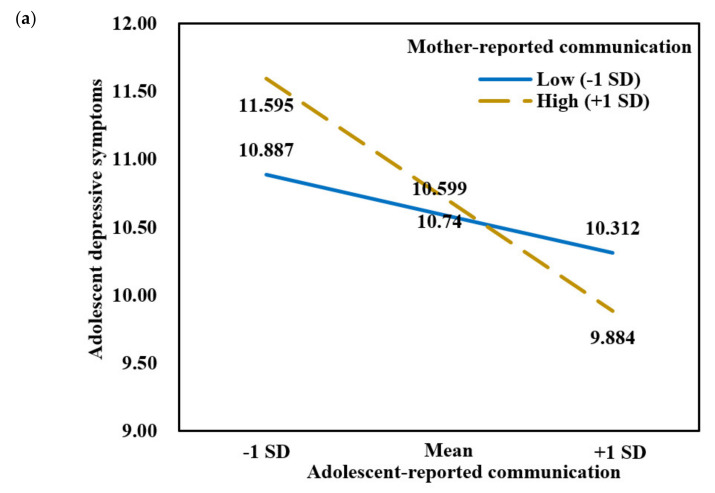

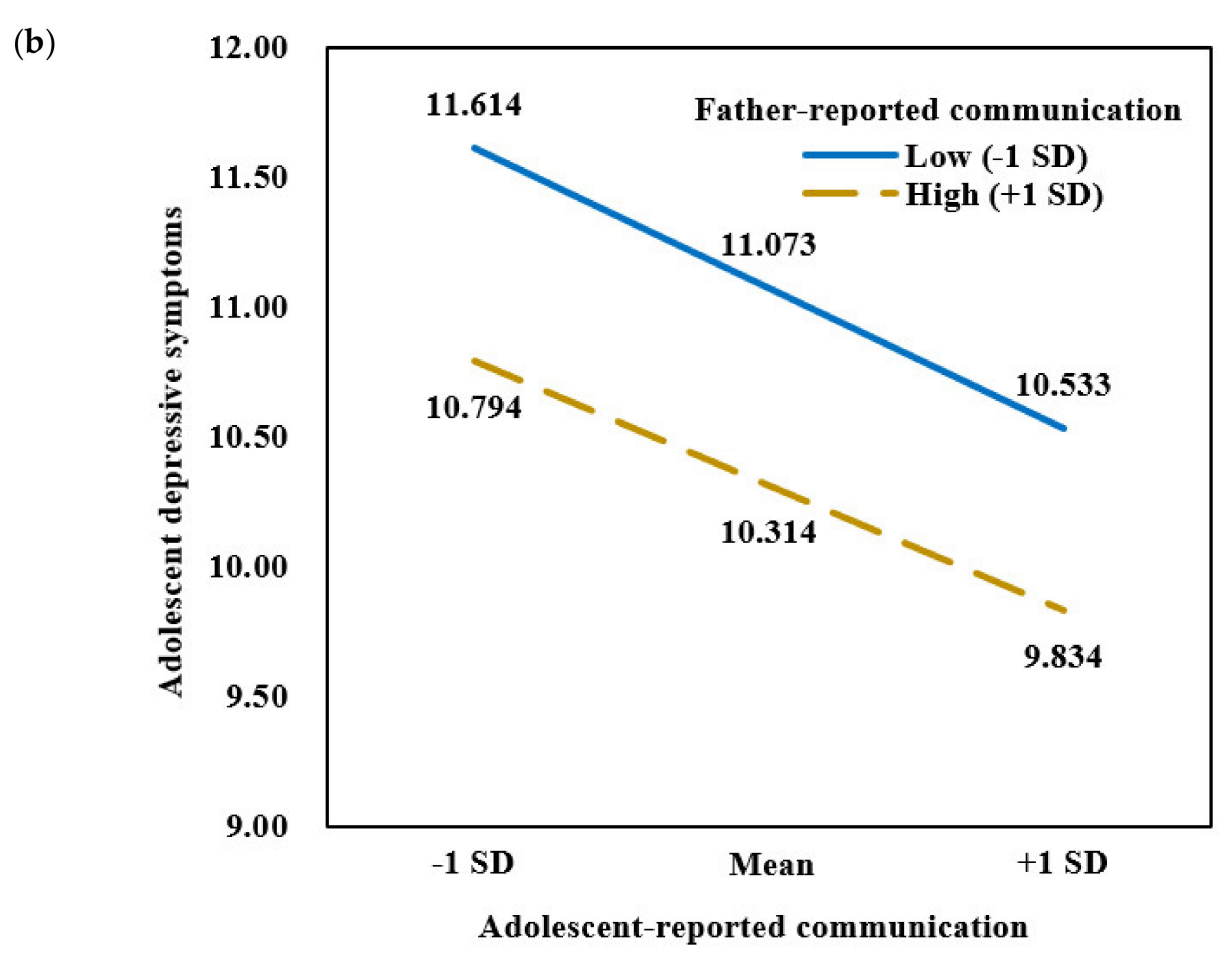
(**a**) Predicted values of depressive symptoms as a function of adolescent-reported communication at high and low levels of mother-reported communication among adolescents in single-mother families (*N* = 1362). (**b**) Predicted values of depressive symptoms as a function of adolescent-reported communication at high and low levels of father-reported communication among adolescents in single-father families (*N* = 443).

Regarding single-mother families, the interaction between mother-reported and adolescent-reported mother-adolescent communication on adolescent depressive symptoms was significant, *b* = −0.287, *SE* = 0.127, *t* = −2.256, *p* = 0.024, 95% *CI* = [−0.537, −0.037]. The findings are presented in Table 5. Simple slope analysis showed that when adolescent perceived low mother-adolescent communication, adolescent depressive symptoms were higher when mother-reported mother-adolescent communication was at high level *b* = −0.869, *SE* = 0.173, *t* = -5.021, *p* < 0.001, 95% *CI* = [−1.209, −0.530] than at low levels of mother-reported communication, *b* = −0.292, *SE* = 0.179, *t* = −1.634, *p* = 0.102, 95% *CI* = [−0.643, 0.059]. In other words, high mother-perceived mother-child communication but low adolescent-perceived mother-child communication was linked with the highest adolescent depression, whereas high mother-perceived and high adolescent-perceived mother-child communication was linked with the lowest adolescent depression (see Figure 2a). However, no significant interaction effect between father-reported and adolescent-reported father-adolescent communication was found in single-father families (see Figure 2b). In addition, for parent-absent families, the interactions between mother-reported (or father-reported) and adolescent-reported mother-adolescent/father-adolescent communication on adolescent depressive symptoms were not significant. Hypothesis 4 was supported.

## 4. Discussion

The present study makes a pioneer contribution to understanding how congruency or discrepancy between parent-reported and adolescent-reported parental communication influence adolescent depressive symptoms in Chinese intact and non-intact families. First, we investigated the discrepancies in mother-adolescent and father-adolescent communication, and their linkages with adolescent depressive symptoms in intact and non-intact families, by using the nationally representative sample in China. Second, we used polynomial regression analyses to examine the interactive effects of how informant discrepancies would influence early adolescent depressive symptoms in China. This methodology is superior because recent studies showed that polynomial regression analyses could yield more accurate findings than difference scores [63,70]. Third, our findings provide support for the view that parent-child discrepancy in the perceived family process is pathological where parent-child discrepancies in perceived family processes have maladaptive impacts on children’s developmental outcomes.

### 4.1. Discrepancies between Parents and Adolescents on Perceived Parent-Child Communication

Results indicated that parents and adolescents had a different perception of parent-child communication, with adolescents having more negative perceptions than parents in China. The results are consistent with previous studies showing adolescents perceive more negative parent-adolescent communication than did their parents in Western societies [23,29,63]. In the Chinese context, discrepancies between perceptions of parent-adolescent communication may be intensified due to hierarchical decision-making and the lack of emotional expression within the Chinese families [73]. For Chinese parents, they are responsible for educating and nurturing their children, so they are more likely to overestimate their efforts in parent-adolescent communication, whereas Chinese adolescents often demonstrate their autonomy and independence by showing their differences from their parents. In Confucian culture, Chinese adolescents are required to be obedient, so they are less likely to communicate openly on disagreements and conflicts with their parents [74]. Besides, Chinese parents expressed less positive affection to their children compared to American parents [75]. The less affectional communication circumstance may lead to adolescents’ lower evaluations of parent-adolescent communication relative to their parents [55,76]. Additionally, as Chinese fathers showed less responsiveness, warmth, and concern, adolescents reported more negative feelings when communicating with their fathers than did fathers report [77].

Moreover, we found that father-adolescent discrepancies in perceived paternal communication were greater in intact families than non-intact families, whereas mother-adolescent discrepancies in perceived maternal communication were the same in intact families as well as non-intact families. This finding did not fully support Hypothesis 2 which argues that parent-adolescent discrepancies were greater in non-intact families than intact families. The reason may be that fathers perceived lower levels of father-child communication in non-intact families (such as single-father families, parent-absent families) than intact families (two-parent families). This can also be explained by the fact that as mothers are the main socialization agent, their communication with their children is more intense as compared to that of the fathers.

### 4.2. Parent-Adolescent Discrepancies in Perception of the Parent-Child Communication and Adolescent Depressive Symptoms in Two-Parent Families

Results indicated that, for adolescents from two-parent families, the interaction between father-reported and adolescent-reported communication predicted adolescent depressive symptoms. Specifically, adolescent-perceived father-child positive communication was more strongly linked with less adolescent depressive symptoms when father-perceived father-child communication was high than when it was low. In other words, congruence in high adolescent-perceived and father-perceived father-child positive communication was linked with the lowest levels of adolescent depressive symptoms. Moreover, although the interaction was not significant for mother-adolescent communication discrepancies in two-parent families, a similar pattern was found. Specifically, adolescents have fewer depressive symptoms when both the mother and the adolescent perceived high mother-child communication. These results are consistent with previous Western findings that congruence in low adolescent-perceived and parent-perceived parent-child negative interaction was linked with the lowest adolescent depression [64].

In summary, in two-parent families, high parent-reported and high adolescent-reported parent-adolescent communication were associated with the lowest levels of adolescent depressive symptoms. Theoretically, attachment theory and family ecological models can explain this result. Attachment theory argues that adolescents with secure attachment to their parents are less likely to suffer depressive symptoms relative to adolescents with insecure attachment [78]. Adolescents with secure attachment to their parents usually have more positive parent-child communication compared with insecurely attached adolescents [79], thus both securely attached adolescents and their parents may perceive high levels of parent-child communication. Moreover, family ecological models suggest that the father-child relationship, mother-child relationship, and father-mother relationship in two-parent families together influence children’s developmental outcomes. If both parents and adolescents reported high levels of parent-child communication in two-parent families, which suggests family functioning is well, children are less likely to have detrimental developmental outcomes such as depressive symptoms.

### 4.3. Discrepancies in Perception of Parent-Adolescent Communication and Early Adolescent Depressive Symptoms in Single-Parent Families

For adolescents in single-mother families, results indicated that the interaction between adolescent-reported mother-adolescent communication and mother-reported communication predicted adolescent depressive symptoms. Specifically, low adolescent-perceived but high mother-perceived mother-child communication was linked with the highest adolescent depressive symptoms, while congruence of high adolescent-perceived and mother-perceived mother-child communication was linked with the lowest adolescent depressive symptoms. This observation is consistent with previous studies showing that greater informant discrepancies were linked with more internalizing problems [22,41]. This observation is also consistent with the previous finding that congruence between mothers’ and adolescents’ perception of low conflict was linked with fewer adolescent depressive symptoms [70]. The explanation for this finding is that as the mother-child dyad is the cornerstone of single-mother families, mother-child discrepancies in perceived parenting would create conflict and be a stressor for the adolescent child. In Chinese single-mother families, mothers are responsible for raising their children and they usually have higher expectations and involvement for their children. Chinese mothers are also more concerned about closeness and intimacy, which cannot satisfy early adolescents’ needs for more autonomy and individuality [80,81]. Besides, Chinese mothers pay more attention to adolescents’ academic performance than mothers in western culture [82]. Therefore, in Chinese single-mother families, mothers are likely to perceive high mother-adolescent communication, while adolescents might perceive low mother-adolescent communication, so that mothers would ignore adolescent internalizing behaviors and adolescents cannot seek help from others in families. As a result, adolescents would suffer more depressive symptoms.

However, for adolescents in single-father families, results indicated that interaction between adolescent-reported father-adolescent communication and father-reported communication on adolescent depressive symptoms was not significant. This result suggested that congruence of adolescent-perceived and father-perceived low levels of father-child communication was linked with highest adolescent depression, while congruence of adolescent-perceived and father-perceived high levels of father-child communication was linked with lowest adolescent depression. This result may be explained by low attachment and low bonding between children and their fathers in single-father families. In traditional Chinese culture, children usually have low attachment and bonding with their fathers due to less emotional expression in father-child communication [51]. Moreover, in Chinese single-father families, as fathers need to spend more time on earning money to support the family, they have less time to communicate with their children. Previous studies showed that a high level of father-child communication reduced adolescents’ depressive symptoms [48,83]. As a result, both children and their fathers in single-father families would perceive low levels of father-child communication, in turn, they develop low attachment and low bonding, which could contribute to depressive symptoms.

### 4.4. Theoretical Implications

Our findings have important theoretical implications. Our findings suggest that parent-child discrepancy in the perceived family process is not normative but pathological, and it is not a measurement error, but it has theoretical significance. Specifically, parent-child discrepancies in perceived family processes are associated with maladaptive children’s developmental outcomes, which provide support for the pathological perspective on the nature and impact of parent-adolescent discrepancy in the perceived family process. Furthermore, our findings in the Chinese context suggest the maladaptive impacts of parent-child discrepancy in the perceived family process are universal in different cultures.

### 4.5. Implications for Clinical Practice

Our findings provide information regarding maladaptive impacts of discrepancies between parents and adolescents in the perceived parent-adolescent communication on adolescent developmental outcomes in Chinese two-parent families and single-mother families. First, for adolescents from two-parent families, congruence of adolescent-reported and father- or mother-reported high communication was linked with low levels of depressive symptoms. Thus, for early adolescents in two-parent families, practitioners should pay more attention to adolescents with the congruence of adolescent-reported and parent-reported low parent-adolescent communication. Importantly, intervention programs should be developed to improve the quality of parent-adolescent communication, in particular for those families where there is low father-adolescent communication and larger discrepancies between fathers and adolescents.

Second, among early adolescents from single-mother families, practitioners should be sensitive to adolescents with the discrepancies of adolescent-reported low mother-adolescent communication and mother-reported high communication, because these adolescents were at the highest risk for depressive symptoms. Intervention programs should be designed to improve communication skills and strategies for these adolescents and their mothers in single-mother families. Intervention should focus on raising mothers’ awareness of their perception of mother-child communication as well as discrepancies between mothers and adolescent children.

### 4.6. Limitations of the Study

The present study has several limitations. First, as the present study was a cross-sectional survey, causality between variables cannot be effectively explained. Thus, future studies should employ a longitudinal survey. Second, we only measure parent-driven communication which might have different impacts on adolescent depression compared with child-driven communication [10]. Both parent-driven and child-driven communication should be considered in future studies. Third, we used an established database with fixed measures that may limit the comparisons of other related studies and the clinical application. Fourth, some important factors, such as the size of the family nucleus, the order in which the adolescent occupies the children depending on the number of siblings, have not been controlled. Further studies should discuss these factors. Nevertheless, as families in China are mostly single-child families, this problem should not be great. Fifth, as only one parent (father or mother) in a family participated our study, we could not compare the effects of discrepancies in the mother-adolescent and father-adolescent communication on adolescent depressive symptoms. This should be further studied because family systems theory proposes that the mother-child and father-child relationships are related but different family subsystems, which may be differentially linked with adolescent developmental outcomes [84].

## 5. Conclusions

This study shows that adolescents reported parent-child communication more negatively than did their parents. Father-adolescent discrepancies were also greater in intact families than non-intact families. Moreover, polynomial regression analyses indicated that while there was a significant interactive effect of father-reported and adolescent-reported father-adolescent communication on adolescent depressive symptoms in Chinese two-parent families, no significant interaction was found for the mother-adolescent dyad. Besides, adolescent-reported mother-child communication interacted with mother-reported communication on adolescent depressive symptoms in Chinese single-mother families only.

## Figures and Tables

**Table 1:** Distribution of respondents.

Variable	Father-Adolescent Dyads (*N*)	Mother-Adolescent Dyads (*N*)
Family categories		
Two-parent family	5960	6670
Single-mother family	163	1362
Single-father family	443	60
Family with both parents absence	444	275
Total	7010	8367
Gender		
Boys	3860	3871
Girls	3204	4496
Grade		
7th	3524	4536
9th	3486	3831
Mean Age (years)	14.24 (1.25)	14.02 (1.18)

**Table 2:** Means and SDs of all study variables as well as tests of congruency between parent and adolescent reports of parent-adolescent communication.

Variable	Adolescent-Report	Father-Report	Mother-Report	*t*	Cohen’s *d*
	*M (SD)*	*M(SD)*	*M(SD)*		
Total sample					
Depressive symptoms (*n* = 15,377)	10.14 (3.76)				
Mother-adolescent communication (*n* = 8367)	11.11 (2.81)		11.87 (2.54)	−25.62 ***	−0.28
Father-adolescent communication (*n* = 7010)	9.77 (2.85)	11.23 (2.52)		−40.85 ***	−0.54
Two-parent families					
Depressive symptoms (*n* = 12,630)	10.02 (3.74)				
Mother-adolescent communication (*n* = 6670)	11.23 (2.78)		11.99 (2.49)	−22.91 ***	−0.29
Father-adolescent communication (*n* = 5960)	9.82 (2.83)	11.33 (2.49)		−38.83 ***	−0.57
Single-mother families					
Depressive symptoms (*n* = 1525)	10.57 (3.74)				
Mother-adolescent communication (*n* = 1362)	10.70 (2.83)		11.51 (2.62)	−10.94 ***	−0.30
Father-adolescent communication (*n* = 163)	9.31 (2.65)	11.24 (2.28)		−8.27 ***	−0.78
Single-father families					
Depressive symptoms (*n* = 503)	10.65 (3.78)				
Mother-adolescent communication (*n* = 60)	9.97 (3.00)		11.62 (2.56)	−5.00 ***	−0.60
Father-adolescent communication (*n* = 443)	9.52 (3.08)	10.58 (2.68)		−7.19 ***	−0.37
Families with both parents absent					
Depressive symptoms (*n* = 719)	10.98 (3.89)				
Mother-adolescent communication (*n* = 275)	10.43 (2.85)		10.77 (2.66)	−2.04 *	−0.12
Father-adolescent communication (*n* = 444)	9.45 (2.88)	10.55 (2.63)		−8.08 ***	−0.40

Note. N = 15,377 (including 7010 father-adolescent dyads and 8367 mother-adolescent dyads), * *p* < 0.05, *** *p* < 0.001.

**Table 3:** Comparisons of parent-child discrepancies in perceived parent-adolescent communication between intact families and non-intact families.

	Intact Families *M (SD)*	Non-Intact Families *M (SD)*	*t*	*p*	Cohen’s *d*
Mother-child discrepancy in perceived parent-child communication	0.76 (2.72)	0.76 (2.75)	−0.008	0.994	0.00
Father-child discrepancy in perceived parent-child communication	1.51 (3.01)	1.21 (3.00)	2.98	0.003	0.10

Note. For mother-child discrepancy in perceived parent-child communication, *N* = 6670 in intact families, *N* = 1697 in non-intact families. For father-child discrepancy in perceived parent-child communication, *N* = 5960 in intact families, *N* = 1050 in non-intact families.

**Table 4:** Polynomial regression analyses predicting early adolescent depressive symptoms (father and adolescent (adol.) report father-adolescent communication as predictors).

Predictor	Group 1		Group 2		Group 3		Group 4	
	*b*	*SE*	*b*	*SE*	*b*	*SE*	*b*	*SE*
Family economic status								
Middle income	−0.42 ***	0.12	−0.17	0.38	−0.25	0.62	−0.70 ^†^	0.40
Rich	−0.68 ***	0.23	−1.69 ^†^	0.92	−1.87	1.41	−0.57	0.82
Father education	0.03	0.03	0.04	0.12	0.23	0.21	0.11	0.15
Mother education	−0.07 *	0.03	−0.06	0.12	−0.24	0.23	−0.23 ^†^	0.14
Grade	0.74 ***	0.10	0.12	0.36	0.64	0.56	0.10	0.35
Gender	−0.51 ***	0.10	−0.89 *	0.36	−0.66	0.59	−0.82 *	0.35
Adol. report communication	−0.51 ***	0.05	−0.47 *	0.19	−0.21	0.33	−0.84 ***	0.20
Father report communication	−0.20 ***	0.06	−0.37 ^†^	0.22	−0.80 *	0.36	−0.80 **	0.23
(Adol. report)^2^	−0.04	0.05	−0.26	0.17	−0.35	0.31	0.59 **	0.19
(Father report)^2^	−0.05	0.05	−0.08	0.15	−0.27	0.27	−0.29 ^†^	0.17
Adol. report × Father report	−0.12 *	0.06	0.03	0.20	0.005	0.36	−16	0.21
Adjust R^2^	0.048		0.056		0.032		0.114	
*N*	5960		443		163		444	

Note. Grade: 0 = 7th grade, 1 = 9th grade. Gender: 0 = girl, 1 = boy. Reference group of Family economic status: poor family. ^†^ *p* < 0.10, * *p* < 0.05, ** *p* < 0.01, *** *p* < 0.001. Group 1: adolescents in two-parent families (*N* = 5960); Group 2: adolescents in single-father families (*N* = 443); Group 3: adolescents in single-mother families (*N* = 163); Group 4: adolescents in parent-absent families (*N* = 444).

**Table 5:** Polynomial regression analyses predicting early adolescent depressive symptoms (mother and adolescent (adol.) report mother-adolescent communication as predictors).

Predictor	Group 1		Group 2		Group 3		Group 4	
	*b*	*SE*	*b*	*SE*	*b*	*SE*	*b*	*SE*
Family economic status								
Middle income	−0.83 ***	0.14	0.86	1.66	−0.54 *	0.23	−1.01 ^†^	0.57
Rich	−0.90 ***	0.21	1.68	2.36	−1.14 ^†^	0.60	1.55	1.34
Father education	0.09 **	0.03	0.07	0.35	−0.07	0.07	−0.16	0.17
Mother education	−0.07 *	0.03	−0.64 ^†^	0.37	0.00	0.07	−0.01	0.17
Grade	0.72 ***	0.09	2.32 *	1.14	0.61 **	0.20	1.01 *	0.49
Gender	−0.41 ***	0.09	−1.69	1.17	−0.28	0.20	−0.93 ^†^	0.48
Adol. report communication	−0.60 ***	0.05	0.82	0.92	−0.57 ***	0.12	−0.71 *	0.28
Mother report communication	−0.03	0.06	−0.95	0.95	0.08	0.12	0.41	0.30
(Adol. report)^2^	−0.04	0.05	−0.25	0.74	0.22 ^†^	0.12	0.20	0.28
(Mother report)^2^	0.05	0.05	0.40	0.77	0.21 *	0.10	0.32	0.24
Adol. report × Mother report	−0.06	0.06	−0.97	1.15	−0.29 *	0.13	−0.43	0.32
Adjust R^2^	0.045		0.084		0.038		0.051	
*N*	6670		60		1362		275	

Note. Grade: 0 = 7th grade, 1 = 9th grade. Gender: 0 = girl, 1 = boy. Reference group of Family economic status: poor family. ^†^ *p* < 0.10, * *p* < 0.05, ** *p* < 0.01, *** *p* < 0.001. Group 1: adolescents in two-parent families (*N* = 6670); Group 2: adolescents in single-father families (*N* = 60); Group 3: adolescents in single-mother families (*N* = 1362); Group 4: adolescents in parent-absent families (*N* = 275).

## Data Availability

We use the open data from the China Education Panel Survey which is available online: http://cnsda.ruc.edu.cn/index.php?r=projects/view&id=72810330 (accessed on 20 May 2020).
